# Sense of coherence in Chinese and German students

**DOI:** 10.4102/hsag.v24i0.1151

**Published:** 2019-07-18

**Authors:** Claude-Hélène Mayer, Lynette Louw, Hartmut von der Ohe

**Affiliations:** 1 Department of Management, Rhodes University, Grahamstown, South Africa; 2 Department of Industrial and Organisational Psychology, University of South Africa, Pretoria, South Africa

**Keywords:** health, salutogenesis, sense of coherence, Chinese students, German students

## Abstract

**Background:**

Mental health and salutogenesis are important topics at universities in China and Germany where heightening stress levels in students can be observed.

**Aim:**

The aim of this article is to determine the profile of the salutogenic concept, sense of coherence (SOC), in Chinese and German students to provide new insights into SOC and mental health in Chinese and German students in higher education institutions (HEIs).

**Setting:**

The study was carried out at universities in China and Germany.

**Method:**

A non-experimental, cross-sectional, survey-based research design and convenience sampling was utilised to obtain the sample (*n* = 356). The sample was derived at a selected Chinese (*n* = 255) and a selected German university (*n* = 101). Data were gathered using the 7-point Likert SOC dimension scales based on the Life-Orientation Questionnaire (LOQ) research instrument. The internal consistency levels of the SOC sub-scales were of acceptable levels. Descriptive and inferential statistics were used to analyse the data. Cronbach’s alpha coefficients were calculated to determine the reliability of the LOQ research instrument. General linear modelling techniques.

**Results:**

The results showed the Chinese students scored significantly lower in all three SOC scales than the German students, with the largest practical significant difference in the sub-score of meaningfulness. In general, female Chinese and German students scored higher than their male counterparts. No significant differences could be found between German female and male, and Chinese female and male students.

**Conclusion:**

Conclusions and recommendations for future research and HEI practice are provided.

## Introduction

With increasing globalisation, higher education institutions (HEIs) are in demand to produce competitive and healthy leaders in diverse contexts (Ruben, De Lisi & Gigliotti 2017). However, research has shown that students suffer particularly from stress (Regehr, Glancy & Pitts 2013), burnout, anxiety and depression (Gallego et al. 2014), and mental and physical health problems generally have increased among students in different cultural contexts (Lin & Huang 2012; Schaufeli et al. 2002). Among Chinese students moderate depression is prevalent (Chen et al. 2013), and in the German context, Gumz et al. (2014) have pointed out that students of different degrees show significantly higher psychological and physiological symptoms than the German population in general.

Higher education institutions are constantly challenged by change, internationalisation and innovation (Fan et al. 2017) and are important organisations to promote health and well-being (Dooris, Doherty & Orme 2017). Jonker, Koekemoer and Nel (2015) have called for a growing focus on positive elements in organisations in theory and practice. At the same time, health-promoting universities have been established in many countries, such as China (Xiangyang et al. 2003) and Germany (German Network of Health Promoting Universities 2010), to respond to the question: ‘What keeps people healthy?’ – a question that was introduced into scientific discourse by Antonovsky (1979, 1987). With this question he introduced the concept of salutogenesis, which regards health as an active, dynamic self-regulating process in a human being (Bengel, Strittmatter & Willmann 2001). Generally, an individual’s state of health is largely determined by their attitude towards the world and their own life (Antonovsky 1993b:972), as well as the influence of external factors such as war and starvation. However, if the external factors are comparable, then an individual’s state of health and life orientation depend on his or her cognitive and affective motivational perspective of life. Life orientation, in turn, influences the strength of an individual’s position to utilise the resources available to maintain their health and well-being. This life orientation is called ‘sense of coherence’ (SOC) (Antonovsky 1979), and refers to consistency, congruence and harmony. The more pronounced an individual’s SOC, the healthier they will be and the more quickly they will regain health or remain healthy. Sense of coherence can be further described as being (Antonovsky 1987):

a global orientation that expresses the extent to which one has a pervasive, enduring though dynamic feeling of confidence that (1) the stimuli deriving from one’s internal and external environments in the course of living are structured, predictable, and explicable; (2) the resources are available to one to meet the demands posed by these stimuli; and (3) these demands are challenges worthy of investment and engagement. (p. 19)

Generally, SOC, which is based on the three components, *comprehensibility* (one’s understanding of the world based on the ability to process familiar and unfamiliar stimuli as ordered, structured and consistent), *manageability* (how one copes with challenges and beliefs that challenges can be solved through use of resources at hand) and *meaningfulness* (how one is motivated through the construction of meaning in life – the extent to which life makes sense), is a concept that can support students, teachers and parents in educational contexts to cope with challenges and stay healthy (Adelman & Taylor 1998; Coll & Magnuson 2000).

Previous studies have pointed out that research from a positive psychology perspective^1^ is needed to focus on the resources and coping strategies that effectively help to manage academic stressors in students (Sagone & De Caroli 2014) and academic staff (Mayer, Surtee & Visser 2016). Researchers have focused on exploring students in different university settings (Darling et al. 2007; Kuupelomä’ki & Utriainen 2003; Suraj & Singh 2011; Von Bothmer & Fridlund 2003), particularly because SOC is viewed as enhancing a person’s general health and well-being (Tan et al. 2016), preventing mental illness (Pretorius et al. 2009) and helping individuals to cope within complex, international and intercultural settings (Mayer 2011). Sense of coherence is positively associated with various individual outcomes, such as resilience, as well as mental and physical health (Morrison & Clift 2006). The concept of salutogenesis and its main component of SOC have been researched globally (Becker et al. 2009) and it has been emphasised in educational contexts that a strong SOC is associated with positive performance, achievement, success and the ability to manage conflicts and intercultural communication well in complex contexts (Mayer & Boness 2011). Sense of coherence can be applied on different system levels, such as individual, group, organisational and societal (Eriksson 2017).

This article focuses on SOC in Chinese and German students. Research within the salutogenetic paradigm in China began in 2005 (Dai, Lu & Fu 2017). This article aims to contribute to this increasing interest in SOC in Chinese populations and contexts, which still mainly focuses on mental health in patients and occupational health contexts (Dai et al. 2017). It also contributes to research on SOC which is limited in HEIs in general. At the same time, the academic student exchange between China and Germany has increased to more than 800 German-Chinese collaborations, and Chinese students have become the biggest foreign student group at German universities (DAAD & DZHW 2016). German students’ interest in studying in China is also increasing (Mussenbrock 2014). Therefore, this study aims at exploring and comparing SOC in both groups. This will provide an indication of what to expect in terms of SOC similarities and differences in Chinese and German students who meet increasingly in HEI contexts.

Although SOC is relatively stable over time, it has been pointed out that SOC can change over time (Kähönen et al. 2012; Pakkala et al. 2012; Vastamäki, Moser & Paul 2008), through therapy, counselling, resource enhancement and intervention programmes (Gimpel et al. 2014; Krause & Mayer 2012; Mayer & Boness 2013; Mayer & Krause 2011, 2012, 2013; Tan et al. 2016). This insight provides HEIs with the possibility to develop and/or increase SOC in students during their time in an HEI to prepare them for work life and their career development in the future while focusing on their health and well-being (e.g. Mayer 2014; Mayer & Boness 2011; Savickas et al. 2009).

Studies in the HEI context have shown that SOC influences the levels of perceived stress in students, thereby highlighting that a high SOC correlates with lower levels of perceived stress (Torsheim, Aaroe & Wold 2001), and SOC is also a predictor of the health and well-being of students during adolescents (Togari et al. 2007). Other studies have found that SOC is connected to gender and/or sex (Mayer et al. 2016), healthy life styles (Suraj & Singh 2011) and social support (Nosheen, Naveed Riaz & Batool 2014). A study on Polish students showed that SOC correlates positively with a tendency to health-related behaviour: the higher the SOC, the higher the tendency to display healthy behaviour, such as eating healthy and participating in sports (Binkowska-Bury & Januszewicz 2010). Furthermore, a high SOC in students in Northern India is associated with increased health-promoting lifestyle profiles (Suraj & Singh 2011).

Binkowska-Bury and Januszewicz (2010) highlight that SOC in students is usually characterised by an average level of SOC in their Polish sample (Binkowska-Bury & Januszewicz 2010). A comparative study on SOC among religious and non-religious students from Germany and Poland (Schonder 2016) shows that religious belonging or belief is relevant for SOC: Buddhists score the highest in SOC, correlating with the highest scores in self-efficacy and lowest stress experiences. It is interesting to note that although the religious belonging predefined the SOC of Polish students, national belonging did not influence the SOC scores as such. Previous research on Chinese students in Australia has pointed out that stress positively correlates with SOC and that an increase in manageability and meaningfulness could help to decrease stress and increase well-being in nursing students (Lee & Jun 2013). One study (Chu et al. 2016) shows that a stronger SOC in Chinese students is associated with increased social support, better performance compared to peers, integration at university and satisfaction with the political situation. A study on German students shows that SOC correlates with learning styles in medical students (Burger & Scholz 2014).

Furthermore, SOC in Chinese students was negatively associated with perceived stress and positively with health awareness and the importance of nutrition (Chu et al. 2016). A high SOC is also associated with higher anxiety levels, and students with a high SOC tend to be anxious in their interpersonal interactions, which is often counterbalanced by avoidance and social support-seeking strategies in US and Chinese students (Li 2015). A study comparing SOC in Pakistani and German students emphasises that SOC mediates stress and social support in both student groups and that SOC needs to be increased by improving social network structures (Nosheen et al. 2014). A high SOC in Chinese-American students correlates with the experience of low college challenges and low scores regarding symptoms of depression, while a low SOC was associated with high symptoms of depression and a high number of college challenges (Ying, Lee & Tsai 2007). Only one study could be found that explores subjective health complaints among Chinese and German university students (Chu et al. 2015), highlighting that country-specific interventions are needed to address high stress levels and health complaints. A study that compared SOC in Chinese and German students could not be found.

With regard to sex and/or gender in the HEI context, SOC seems to support women in situations to overcome work challenges (Louw, Mayer & Surtee 2014; Mayer 2011). Sense of coherence studies on female academics showed that low SOC scores correlate with high burnout scores (Bezuidenhout & Cilliers 2010) and that women in comparison to men display lower SOC scores in different cultural contexts (Antonovsky 1987; Lindström & Eriksson 2005; Mayer 2011). However, in HEI research in Sweden, researchers found that the mean scores for SOC in male and female students were similar (Von Bothmer & Fridlund 2003), whereas in a northern Indian university setting, female students showed higher SOC scores than their male counterparts. A study has shown that SOC is mainly shaped by parent–child relationships, followed by positive models of behaviour in the peer group, and classroom and teacher support (Garcia-Moya, Moreno & Rivera 2013). The latter was more important for female than for male students. One study on college students in Shanghai showed that female students had higher SOC scores than male students (Li et al. 2014).

The purpose of this article was to determine the profile of the total SOC scores and the SOC components (three sub-scales) and to analyse SOC and its sub-components for a group of Chinese and German students. It analysed the SOC in Chinese and German students to contribute to a deeper insight on SOC and therefore mental health in Chinese and German students in HEIs.

## Materials and methods

### Research objectives and hypotheses

To achieve the purpose of this article and to better understand the SOC dimensions in a cohort of Chinese and German students in specific sociocultural learning contexts, the following objectives have been stated to:

- determine the profile of SOC sub-scales for the Chinese and German student groups
- determine whether there are differences in scores for each SOC sub-scale between the Chinese and German student groups (Hypothesis 1)
- ascertain whether there are differences in the scores for each SOC sub-scale and the combined Chinese and German female and male students (Hypothesis 2)
- ascertain whether there are differences in the scores for each SOC sub-scale and the sex of the Chinese and German student groups, respectively (Hypotheses 3)
- ascertain whether there are differences in the scores for each SOC sub-scale and the sex within the Chinese and German student groups, respectively (Hypotheses 4).

### Research design and procedure

This quantitative study followed a non-experimental cross-sectional survey based on descriptive research to determine the profiles of the SOC sub-scales, as well as the statistical differences between the SOC sub-scale scores for each of the Chinese and German group of students and sex in one measurement procedure. Given the nature of the research objectives, hypotheses and quantitative date collected, a psychometric research paradigm is followed in this study.

Data were gathered over the period of 2 years in Germany (2013–2014) and in China (2011–2012). A quantitative, self-administered questionnaire-based research method was used. The procedure followed a paper-and-pencil approach.

Non-probability sampling was used in this study. Convenience sampling was utilised to obtain a total workable sample of 356 students of which the Chinese students comprised 71.6% (*n* = 255) and German students comprised 28.4% (*n* = 101). As this is a limited sample of the students attending these HEIs, no generalisability to the broader student population can be inferred.

The Chinese student populations comprised 301 students, with 256 responses. One response was excluded in the data cleaning process, resulting in 255 usable responses for the data analysis in this article. The response rate is 85%. The response rate in the German sample is 100%.

The student groups at the Chinese university were studying Event and Tourism Management at undergraduate level, while the student groups at the German university were studying Management at Masters level. However, the age difference between Bachelor and Master students, usually, does not exceed 1–2 years. Surveys were distributed as hard copies only. The data were captured and processed using a standard statistical package (SPSS V.24 2016).

Usual ethical considerations, such as anonymity, confidentiality, voluntary participation, informed consent and the right of the participant to withdraw from the research study at any point in time, were followed. The universities at which the research was conducted agreed to the conduct of the research study. Ethical clearance was provided from the European University Viadrina, Frankfurt (Oder), Germany.

### Research instrument

The questionnaire used in this study comprised two sections. Section A gathered biographical information. Section B included the 7-point Likert SOC dimension sub-scales of comprehensibility, manageability and meaningfulness, referred to as the 29-item Life-Orientation Questionnaire (LOQ) (Antonovsky 1979) research instrument. Of the 29 items, 13 were phrased negatively and required reverse scoring so that a high score in the questionnaire represents a high SOC (Antonovsky 1993a). Eleven items measured comprehensibility (e.g. When you talk to people, do you have the feeling that they don’t understand you?), 10 items measure manageability (e.g. Has it happened that people whom you counted on disappointed you?) and eight items measure meaningfulness (e.g. Do you have the feeling that you don’t really care about what goes on around you?).

Antonovsky (1993b) found the Cronbach’s alpha coefficients (Cronbach 1951) of the 29-item LOQ varied between 0.85 and 0.90 for Western populations. Research on SOC in Germany (Bengel, Wirtz & Zwingmann 2008) found that the Cronbach’s alpha coefficients score for the total SOC scale was 0.92, and more specifically 0.79 for comprehensibility, 0.81 for manageability and 0.86 for meaningfulness. The reliability of the research instrument scales used in this study was, therefore, regarded as acceptable.

### Data analyses

For the quantitative statistical analysis, IBM SPSS Statistics 24 (SPSS 2016) was used to calculate descriptive and inferential statistics. Cronbach’s alpha coefficients were calculated to determine the reliability of the LOQ research instrument. General linear modelling techniques (Hair et al. 2010) were used to analyse the data for possible interactions between the various variables.

*T-test for independent groups* (Field 2006) was used to determine whether there were any significant differences between the mean scores for the different sub-scales of comprehensibility, manageability, meaningfulness and the SOC total scores as metric dependent variables and between the Chinese and German students, the male and female students, and the various possible other sub-groupings formed by the non-metric variables of sex and nationality. As the above *t* test assumes that the samples were drawn from a population with equal variances, SPSS tests for this and when the Levene’s test for equality of variances is significant (*p* < 0.05), homogeneity of variance cannot be assumed and the alternate reported results are used (Pallant 2005:198). Furthermore, Cohen’s *d* (Cohen 1992) was utilised to determine effect sizes, which are an indication of whether the statistical significant differences, which were found in scores on a sub-scale between sub-groupings, had any practical significance and were not only the result of statistical artefacts, such as sample size or the statistical significance criterion level (alpha-level; Cohen 1992). The effect size can also be seen as an indication of the non-overlap of the two sub-groups’ scores. According to Gravetter and Wallnau (2011:233), Cohen’s *d* values of between 0.2 and 0.5 indicate a difference of small practical significance, values between 0.5 and 0.8 reflect differences of moderate practical significance and values greater than 0.8 indicate a difference of large practical significance.

### Ethical considerations

Ethical clearance for the collection of data for this study was provided from the European University Viadrina, Frankfurt (Oder), Germany. Data were gathered over the period of 2 years in Germany (2013–2014) in China (2011–2012). Permission was granted verbally by the Chinese and the German universities participating in the study. At the time of collecting this data, only verbal permission was necessary to collect the data at the said universities in China and Germany.

## Results

With regard to the biographical data of students, the only common variable available and of interest for analysis was sex.

### Biographical information

Of the 356 respondents, 71.6% (*n* = 255) were Chinese students and 28.4% (*n* = 101) were German students. The majority of students were males (232, 67.2%) compared to females (113, 32.8%), as shown in Table 1. In the Chinese sample, more than 80% (*n* = 202) were male students, while only 43 (17.6%) were female students. Conversely, in the German sample, the female students represented the majority (70, 70%) compared to male students (30, 30%).

**TABLE 1:** Gender distribution of sample.

Nationality	Gender	Count	Valid percentage (%)	Percentage of total female and male (%)	Percentage of total (%)
China	Female	43	17.6	38.1	12.5
	Male	202	82.4	87.1	58.6
Germany	Female	70	70.0	61.9	20.3
	Male	30	30.0	12.9	8.7
**Total**	**Female**	**113**	**32.8**	**-**	**-**
	**Male**	**232**	**67.2**	**-**	**-**

Missing: China *n* = 10; Germany *n* = 1.

As shown in Table 2, the overall Cronbach’s alpha coefficient of the total SOC scale is 0.817, with scores of 0.775 and 0.815 (all above the recommended level of 0.70; Hair et al. 2010) achieved for the Chinese and German student groups, respectively. As such, the overall Cronbach’s alpha coefficient score values in this study indicate a high internal consistency and the questionnaire is deemed as being reliable. More specifically, the overall Cronbach’s alpha coefficient score for comprehensibility was 0.607; manageability, 0.648; and meaningfulness, 0.765. Even though the two sub-scales of comprehensibility and manageability were within the lower limit of acceptability for the internal consistency (0.60–0.70) (Hair et al. 2010), they are deemed as being acceptable at the lower limit in exploratory research, while in the Chinese sample the internal consistency varies immensely. For the Chinese student sample, the Cronbach’s alpha coefficient score for comprehensibility (0.551) is below the lower limit score of 0.60, manageability scored 0.607 (lower limit but acceptable) and meaningfulness scored 0.719 (acceptable).

**TABLE 2:** Reliability statistics for the sense of coherence dimension sub-scales.

SOC sub-scales	Chinese students: Cronbach’s α	German students: Cronbach’s α	Total sample: Cronbach’s α	Number of items
Comprehensibility	0.551	0.699	0.607	11
Manageability	0.607	0.680	0.648	10
Meaningfulness	0.719	0.671	0.765	8
LOQ total	0.775	0.815	0.817	29

SOC, sense of coherence; LOQ, Life-Orientation Questionnaire.

In the case of the German student sample, the Cronbach’s alpha coefficient scores for all the SOC sub-scales are very close to the acceptable level of 0.70, as shown in Table 2.

In responding to the objectives and hypotheses posed in this study, the findings presented in the following sections are based on the information provided in Table 3. It should be noted that the number of responses for each of the analyses is indicated by the *N* value in Table 3. Because not all the respondents responded to each statement, the *N* value varies from the total number of respondents, namely 255 Chinese and 101 German students. A similar variation is also evident for the analyses based on sex.

**TABLE 3:** Descriptive and comparative statistics.

Scale	Grouping	Group statistics				Effect size		Levene’s test for equality of variances		Independent samples *t* test for equality of means						
		*N*	Mean	Standard deviation	Standard error of mean					*t*	df	Sig. (two-tailed)	Mean difference	Standard error of difference	95% confidence interval of the difference	
						Cohen’s *d*	Standard	*F*	Sig.						Lower	Upper
Comprehensibility	Chinese	252	45.22	6.98	0.44	-	-	-	-	-	-	-	-	-	-	-
	German	100	49.36	7.73	0.77	0.56	M	0.307	0.580	−4.865	350	0.000	−4.142	0.851	−5.816	−2.467
Manageability	Chinese	248	47.58	7.05	0.45	-	-	-	-	-	-	-	-	-	-	-
	German	101	52.47	6.34	0.63	0.73	M	1.075	0.301	−6.038	347	0.000	−4.885	0.809	−6.476	−3.294
Meaningfulness	Chinese	248	38.07	6.98	0.44	-	-	-	-	-	-	-	-	-	-	-
	German	101	45.30	5.11	0.51	1.18	L	15.616	0.000	−10.708	251†	0.000	−7.224	0.675	−8.553	−5.896
SOC score	Chinese	246	131.01	16.07	1.02	-	-	-	-	-	-	-	-	-	-	-
	German	100	146.95	15.10	1.51	1.02	L	0.504	0.478	−8.509	344	0.000	−15.942	1.874	−19.627	−12.257
Comprehensibility	Female all	110	48.47	7.92	0.76	-	-	-	-	-	-	-	-	-	-	-
	Male all	231	45.60	6.94	0.46	−0.39	S	1.963	0.162	3.408	339	0.001	2.871	0.842	1.214	4.528
Manageability	Female all	109	50.50	6.74	0.65	-	-	-	-	-	-	-	-	-	-	-
	Male all	229	48.43	7.36	0.49	−0.29	S	0.111	0.739	2.491	336	0.013	2.077	0.834	0.436	3.717
Meaningfulness	Female all	109	43.01	6.95	0.67	-	-	-	-	-	-	-	-	-	-	-
	Male all	229	38.99	7.14	0.47	−0.57	M	0.295	0.587	4.882	336	0.000	4.022	0.824	2.402	5.643
SOC score	Female all	107	142.16	17.89	1.73	-	-	-	-	-	-	-	-	-	-	-
	Male all	228	133.08	16.36	1.08	−0.53	M	1.664	0.198	4.596	333	0.000	9.080	1.976	5.193	12.967
SOC score	Female all	107	142.16	17.89	1.73	-	-	-	-	-	-	-	-	-	-	-
	Male all	228	133.08	16.36	1.08	−0.53	M	1.664	0.198	4.596	333	0.000	9.080	1.976	5.193	12.967
Comprehensibility	Chinese female	41	45.78	6.44	1.01	-	-	-	-	-	-	-	-	-	-	-
	German female	69	50.07	8.32	1.00	0.58	M	1.786	0.184	−2.836	108	0.005	−4.292	1.514	−7.292	−1.292
Manageability	Chinese female	39	47.49	6.26	1.00	-	-	-	-	-	-	-	-	-	-	-
	German female	70	52.19	6.44	0.77	0.74	M	0.002	0.968	−3.689	107	0.000	−4.699	1.274	−7.224	−2.173
Meaningfulness	Chinese female	39	37.85	7.13	1.14	-	-	-	-	-	-	-	-	-	-	-
	German female	70	45.89	4.92	0.59	1.31	L	5.895	0.017	−6.260	59†	0.000	−8.040	1.284	−10.610	−5.469
SOC score	Chinese female	38	131.74	16.47	2.67	-	-	-	-	-	-	-	-	-	-	-
	German female	69	147.90	16.03	1.93	0.99	L	0.140	0.709	−4.943	105	0.000	−16.162	3.270	−22.645	−9.679
Comprehensibility	Chinese male	201	45.28	7.01	0.49	-	-	-	-	-	-	-	-	-	-	-
	German male	30	47.73	6.15	1.12	0.37	S	1.675	0.197	−1.812	229	0.071	−2.450	1.352	−5.114	0.214
Manageability	Chinese male	199	47.72	7.27	0.52	-	-	-	-	-	-	-	-	-	-	-
	German male	30	53.10	6.28	1.15	0.79	M	0.967	0.327	−3.841	227	0.000	−5.376	1.400	−8.135	−2.618
Meaningfulness	Chinese male	199	38.22	7.06	0.50	-	-	-	-	-	-	-	-	-	-	-
	German male	30	44.07	5.43	0.99	0.93	L	5.236	0.023	−5.266	45†	0.000	−5.846	1.110	−8.081	−3.610
SOC score	Chinese male	198	131.29	16.09	1.14	-	-	-	-	-	-	-	-	-	-	-
	German male	30	144.90	13.01	2.38	0.93	L	2.812	0.095	−4.417	226	0.000	−13.612	3.082	−19.685	−7.539
Comprehensibility	Chinese female	41	45.78	6.44	1.01	-	-	-	-	-	-	-	-	-	-	-
	Chinese male	201	45.28	7.01	0.49	−0.07	S	0.155	0.694	0.419	240	0.676	0.497	1.186	−1.839	2.833
Manageability	Chinese female	39	47.49	6.26	1.00	-	-	-	-	-	-	-	-	-	-	-
	Chinese male	199	47.72	7.27	0.52	0.03	S	0.269	0.604	−0.190	236	0.850	−0.236	1.246	−2.691	2.218
Meaningfulness	Chinese female	39	37.85	7.13	1.14	-	-	-	-	-	-	-	-	-	-	-
	Chinese male	199	38.22	7.06	0.50	0.05	S	0.205	0.651	−0.303	236	0.762	−0.375	1.239	−2.815	2.065
SOC score	Chinese female	38	131.74	16.47	2.67	-	-	-	-	-	-	-	-	-	-	-
	Chinese male	198	131.29	16.09	1.14	−0.03	S	0.087	0.768	0.157	234	0.875	0.449	2.861	−5.187	6.085
Comprehensibility	German female	69	50.07	8.32	1.00	-	-	-	-	-	-	-	-	-	-	-
	German male	30	47.73	6.15	1.12	−0.32	S	3.573	0.062	1.383	97	0.170	2.339	1.691	−1.017	5.696
Manageability	German female	70	52.19	6.44	0.77	-	-	-	-	-	-	-	-	-	-	-
	German male	30	53.10	6.28	1.15	0.14	S	0.284	0.596	−0.656	98	0.514	−0.914	1.394	−3.681	1.853
Meaningfulness	German female	70	45.89	4.92	0.59	-	-	-	-	-	-	-	-	-	-	-
	German male	30	44.07	5.43	0.99	−0.35	S	0.218	0.642	1.642	98	0.104	1.819	1.108	−0.380	4.018
SOC score	German female	69	147.90	16.03	1.93	-	-	-	-	-	-	-	-	-	-	-
	German male	30	144.90	13.01	2.38	−0.21	S	2.938	0.090	0.903	97	0.369	2.999	3.322	−3.594	9.592

SOC, sense of coherence.

† , Equal variances not assumed: ‘*t*-test performed with separate variance estimates rather than pooled estimate because the Levene’s test detected significant differences in the variations between the two groups’ (Hair, J., Black, W., Babin, B. & Anderson, R., 2010, *Multivariate data analysis*, 7th edn., Pearson, Upper Saddle River, NJ, p. 283).

### Profile of the sense of coherence sub-scales for the Chinese and German student groups

As can be seen from Table 3, in the case of the Chinese students, manageability received the highest mean score of 47.58, followed by comprehensibility (45.22) and meaningfulness (mean score 38.07). A similar finding was found in the case of the German students: manageability also received the highest mean score of 52.47, followed by comprehensibility (49.36) and meaningfulness (mean score 45.30). Overall, the Chinese students scored lower (mean score 131.01) on all the SOC sub-scales than the German students (mean score 146.95).

### Differences in the scores for each sense of coherence sub-scale between the Chinese and German student groups

When comparing the Chinese student group by combining female and male students with the combined female and male German student group, the Chinese student group scored significantly lower than the German student group on all the SOC sub-scales (*p* < 0.000). This difference has the largest practical significance in the case of the meaningfulness sub-scale [*t*(336) = −10.708, *p* < 0.000, *d* = 1.18], although equality of population variances could not be assumed as *Levene’s test for equality of variances* (SPSS 2016) indicated a violation of the assumption of homogeneity of variances (see footnote Table 3). In the case of comprehensibility [*t*(350) = −4.865, *p* < 0.000, *d* = 0.56] and manageability [*t*(347) = −6.038, *p* < 0.000, *d* = 0.73], the differences were statistically significant, but only of moderate practical significance. The total SOC scores (the overall score for all the sub-scales) also had a large practical significance [*t*(251) = −8.509, *p* < 0.000, *d* = 1.02], but this is, in all probability, partially an effect of the items, also representing the meaningfulness sub-scale. No support was found for Ho^1^ as there were differences in the scores for each SOC sub-scale between the Chinese and German student groups. It should be noted that the difference has the largest practical significance in the case of the meaningfulness sub-scale.

### Differences in the scores for each sense of coherence sub-scale and the combined Chinese and German female and male students

By combining the Chinese and German female male students, disregarding the differences in nationality, the female students scored significantly higher than the male students on all sub-scales, as shown in Table 3. However, it should be noted that although significant, the practical significance for comprehensibility [*t*(339) = 3.408, *p* < 0.001, *d* = −0.39] and manageability [*t*(336) = 2.491, *p* < 0.013, *d* = −0.29] was small and moderate in the case of meaningfulness [*t*(336) = 4.882, *p* < 0.000, *d* = −0.57]. A similar trend is found for the total SOC score: on average female students (*M* = 142.16, standard error [s.e.] = 1.73) scored higher than male students (*M* = 133.08, s.e. = 1.08). Even though the overall SOC score and sex difference was significant [*t*(333) = 4.596, *p* < 0.000, *d* = 0.53], because of the moderate-sized effect, the practical significance was moderate. Because there were differences in the scores for each SOC sub-scale between the combined Chinese and German female and male student groups, no support for Ho^2^ was found.

### Differences between sense of coherence sub-scales and sex of Chinese and German student groups

- Ho^3^: There are no differences in the scores for each SOC sub-scale.
- Ho^3.1^: There are no differences in the scores for Chinese and German female students.
- Ho^3.2^: There are no differences in the scores for Chinese and German male students.

In determining whether there was a difference between the SOC sub-scales and sex of Chinese and German students, Chinese female students scored significantly lower than German female students on all the SOC sub-scales (*p* < 0.000). The meaningfulness sub-scale [*t*(59) = −6.260, *p* < 0.000, *d* = 1.31] had the highest effect size (once again equal variances not assumed), indicating a large practical significance. Likewise, the total SOC score [*t*(105) = −4.943, *p* < 0.000, *d* = 0.99] indicated a large practical significance. Comprehensibility [*t*108*)* = −2.836, *p* < 0.005, *d* = 0.58] and manageability [*t*(107) = −3.689, *p* < 0.000, *d* = 0.74] were statistically significantly different, but only moderately practically significant. Support could, thus, not be found for Ho^3.1^ as the Chinese female students scored significantly lower than German female students on all the SOC sub-scales.

The same trend was evident in the case of male students because the Chinese male students scored significantly lower than the German male students on all the SOC sub-scales. However, in the case of the comprehensibility sub-scale [*t*(229) = −1.812, *p* < 0.071, *d* = 0.37], there is no statistical significant difference on the 0.05 level, but from a theoretical perspective it is important to acknowledge that on the less stringent *p* < 0.10 level there is a significant statistical difference. However, in the latter case the effect size was also small, indicating no real significant practical differences. In contrast, both total SOC [*t*(226) = −4.417, *p* < 0.000, *d* = 0.93] and meaningfulness [*t*(45) = −5.266, *p* < 0.000, *d* = 0.93] were not only significantly different but also represented a large-sized effect with a large practical significance. In the case of manageability [*t*(227) = −3.841, *p* < 0.000, *d* = 0.79], there was a moderate practical significance. As such, support could, thus, not be found for Ho^3.2^ as the Chinese male students scored significantly lower than German male students on all the SOC sub-scales.

### Differences in the scores for each sense of coherence sub-scales and sex within the Chinese and German student groups

In ascertaining whether there were differences in the scores for each SOC sub-scales and sex within the Chinese and German student groups, no significant differences were found between each of the SOC sub-scales and the Chinese and German female and male student groups, as shown in Table 3 (*p >* 0.10). In all instances, the practical significance is small. Likewise, no significant differences were found in the total SOC scores for the Chinese and German student groups with a small practical significance [*t*(234) = −0.768, *p* < 0.875, *d* = 1.14] and [*t*(97) = −0.090, *p* < 0.369, *d* = 2.38], respectively. This means that there were no significant sex differences within the Chinese and German student groups; consequently Ho^4.1^ and Ho^4.2^ are supported.

## Discussion

This study contributes to determining the profile of SOC sub-scales in Chinese and German students (Dai et al. 2017) to address the research void in SOC and mental health of these specific groups (Ruben et al. 2017). The sense of urgency is driven by SOC, providing a strong foundation for mental health and well-being, which is needed for students in both countries to manage symptoms of stress and depression (Chen et al. 2013; Gumz et al. 2014). In addition, this research provides new information for HEIs in terms of SOC scores of Chinese students who are the biggest group of foreign students in Germany (DAAD & DZHW 2016).

Overall, the Chinese students scored lower on all the SOC sub-scales than the German students and these differences in scores between the groups were statistically significant. Sense of coherence influences students’ stress levels, lifestyles, health awareness and nutrition (Chu et al. 2016) and the manner in which they relate to social support and network structures (Nosheen et al. 2014), which was not studied in this research, but should be addressed and compared in future studies to explore SOC and healthy lifestyles in these student groups, especially in the case of the Chinese students. However, this finding is very important with regard to students studying abroad. Previous research has shown that expats score lower in SOC than managers who are anchored in their sociocultural environment in organisations (Mayer 2011). If Chinese students with lower SOC scores than German students study at German universities and these students become foreign students at German universities, it might be assumed that the SOC scores might decrease even more when studying in a foreign university context.

Based on the descriptive statistical analyses, in the case of both the Chinese and German students, manageability received the highest mean score, followed by comprehensibility and meaningfulness. This means that the ability to cope with challenges is the most important component for the students and seemingly the most developed in this sample of students, which corresponds with previous studies (Mayer 2011), indicating that for German managers, manageability is also the highest and most important component of SOC, while German managers scored very low in meaningfulness, as also found in this study for both groups of students. Furthermore, the findings show that the lower scores in meaningfulness of Chinese students in this study might relate to the high scores in depression in Chinese students in other studies (Chen et al. 2013; Ying et al. 2007). It should also be noted that statistically significant differences were found for all the SOC sub-scales between the two groups. Despite the lower mean scores for meaningfulness by both groups of students, meaningfulness was the SOC sub-scale that showed the most practical significant difference between Chinese and German students, while manageability was of moderate practical significant difference.

This study further contributes to research on sex, gender and SOC, as recommended in Mayer et al. (2016), who recommended to further investigate the SOC and gender in HEIs. In combining the Chinese and German female and male students, disregarding the differences in nationality, the female students scored significantly higher than the male students on all SOC sub-scales. Although meaningfulness was of moderate practical significance, the other SOC sub-scales were not practically significant. This finding stands partly in contrast to previous research findings in which men scored higher (Antonovsky 1987; Lindström & Eriksson 2005; Mayer 2011) or SOC scores were similar for both genders (Von Bothmer & Fridlund 2003).

This finding supports previous findings from a northern Indian university in which female students scored significantly higher than male students (Garcia-Moya et al. 2013). Furthermore, these findings might support the assumption that SOC scores with regard to sex and/or gender are strongly dependent on the contextual concepts and values, and their broader sociopolitical and cultural context (Antonovsky 1979; Chu et al. 2016; Mayer 2011). How sex and/or gender influence SOC should be further established in follow-up studies.

In determining whether there was a difference between the SOC sub-scales and sex of Chinese and German students, overall the Chinese female students scored significantly lower than German female students on all the SOC sub-scales, with meaningfulness being of large practical significance. The same trend was evident in the case of male students because the Chinese male students scored significantly lower than the German male students on all the SOC sub-scales. However, in their case there was no real significant practical difference in all the SOC sub-scales. How gender concepts are influenced by the sociocultural context and how gender concepts influence the difference in SOC with regard to sex need further exploration.

In ascertaining whether there were differences in the scores for each SOC sub-scale and gender within the Chinese and German student groups, no significant differences were found between each of the SOC sub-scales and the Chinese and German female and male student groups. In all instances, the practical significance is small.

### Limitations

The results need to be viewed and interpreted against this study’s limitations. The first limitation relates to the limited explorative nature of the research as the researchers had access to only one set of data on Chinese and German students in a particular point in time over 2 years. Secondly, the relatively small sample sizes limited the type of statistical analysis that could be used to analyse the data. Thirdly, given the nature of the biographical information of the sample, a bias is present because all students were studying management-related courses. Fourthly, the researchers only had access to students who studied different management-related subjects and degrees that need to be considered reading the data. It is therefore important to further explore the extent to which the types of courses and/or degrees as well as the national origin of participants influence the results.

Finally, the sample was not randomly selected nor randomly assigned to the groups, which can be seen as another limitation of the study.

Despite the fact that the Cronbach’s alpha coefficient scores for comprehensibility (0.551) for the Chinese students were below the traditional lower limit score of 0.60, the scores were included for the data analysis in this study as possible theoretical insights should not be sacrificed. It should be noted that although comprehensibility for the Chinese group was included in the analysis, it was of a low or no practical significance.

## Conclusion, future research and practical implications

The results show differences in SOC scores between Chinese and German students and with regard to sex and gender concepts. The study indicates that although manageability scores were the highest in Chinese and German students, which support them to cope with everyday challenges, meaningfulness was the most practically significant.

Future research should focus on comparing SOC in Chinese and German students across disciplines and at finding explanations to respond to the question why these results are shown. This research could support further sociocultural and contextual understanding of differences and similarities in SOC of both samples. These future findings could be of help in intercultural encounters in different HEIs and work contexts. Future studies should compare SOC in students studying in their home countries and studying abroad to determine if and how the change in context influences SOC scores. Furthermore, more research is needed to explore SOC in students in different cultural contexts in relation to their health and well-being, their healthy lifestyle behaviour and the influence on managerial abilities and leadership competencies. This should be predominantly important for management students.

It would be important to respond to the question of why there are differences in SOC scores across Chinese and German male and female students. Despite the low mean scores in meaningfulness for both groups of students, the understanding of meaningfulness between these two groups of students was significantly different. Future research should further explore the reasons for the low scores in meaningfulness, gain a better understanding of why meaningfulness is significantly different between the two groups and interlink SOC research with research on religious belonging or belief and spirituality. It is also of further interest why Chinese students scored significantly lower in meaningfulness than German students and what aspects build meaningfulness in their (student) life. In addition, the impact that high and low SOC scores have on performance, success, coping and physical health in Chinese and German students and how SOC impacts when Chinese and German students interact in German and in Chinese HEI contexts should be assessed.

On a practical note, HEIs in Germany and China should increase their awareness with regard to SOC in students (as well as staff) and their impact on management students’ education at HEI levels in terms of comprehensibility, manageability and meaningfulness. If HEIs internationally aim at creating healthy learning environments, they need to take SOC on individual and organisational levels into account and develop systemic-oriented health programmes that support the development and increase of SOC on different (HEI) levels. To create healthy, strengthening and empowering learning environments to prepare, particularly management, students for future global leadership positions, HEIs need to develop complex systems to develop SOC and mental health in students. This will definitely be a future challenge in HEI contexts and certainly needed to prepare future employees for demanding professional contexts in which they need to be aware of their mental health and well-being.

Because Chinese students score lower in SOC than German students, German HEIs should focus particularly on supporting Chinese exchange students in understanding the German HEI environment (to strengthen their sense of comprehensibility), helping them to cope during their exchange programmes (manageability) and aiming at exploring meaningfulness in students in general throughout their time at HEIs to support them in coping in the new context.

## Footnotes

1 Salutogenesis and SOC are defined concepts in the positive psychology paradigm (Delle Fave 2006; Eriksson & Lindström 2011; Joseph & Sagy 2017; Mayer 2011).
